# Evaluation of random forests performance for genome-wide association studies in the presence of interaction effects

**DOI:** 10.1186/1753-6561-3-s7-s64

**Published:** 2009-12-15

**Authors:** Yoonhee Kim, Robert Wojciechowski, Heejong Sung, Rasika A Mathias, Li Wang, Alison P Klein, Rhoshel K Lenroot, James Malley, Joan E Bailey-Wilson

**Affiliations:** 1 National Human Genome Research Institute, National Institutes of Health, 333 Cassell Drive, Baltimore, MD 21224, USA; 2 Johns Hopkins University School of Medicine, 733 North Broadway, Baltimore, Maryland 21205, USA; 3 National Institute of Mental Health, National Institutes of Health, 6001 Executive Boulevard, Bethesda, Maryland 20892-9663, USA; 4 Center for Information Technology, National Institutes of Health, 10401 Fernwood Road, Bethesda, Maryland 20817, USA

## Abstract

Random forests (RF) is one of a broad class of machine learning methods that are able to deal with large-scale data without model specification, which makes it an attractive method for genome-wide association studies (GWAS). The performance of RF and other association methods in the presence of interactions was evaluated using the simulated data from Genetic Analysis Workshop 16 Problem 3, with knowledge of the major causative markers, risk factors, and their interactions in the simulated traits. There was good power to detect the environmental risk factors using RF, trend tests, or regression analyses but the power to detect the effects of the causal markers was poor for all methods. The causal marker that had an interactive effect with smoking did show moderate evidence of association in the RF and regression analyses, suggesting that RF may perform well at detecting such interactions in larger, more highly powered datasets.

## Background

Random forests (RF) [1] is one of a broad class of machine learning methods that are able to deal with large-scale data without precise model specification: it is massively nonparametric. It performs random searches through feature space and data space, the latter by using bootstrap sampling. It generates multiple recursively partitioned classification trees (the exact number determined by the analyst), called a "forest". RF has gained attention as a method that may be useful for detecting associations when there are large numbers of predictor variables to be evaluated, such as single-nucleotide polymorphism (SNP) loci in a genome-wide association study (GWAS). It has also been suggested that RF may perform better than other methods when the causative loci have minimal marginal effects but larger interaction effects [2-6]. The calculation of importance values and the very local nature of the classification in each tree allows RF to automatically evaluate gene-gene and gene-environmental interactions [5] without assuming complex models or explicitly testing all possible interactions. Many GWAS test each locus under the generally implausible assumption of complete or weak statistical independence across all loci. RF evaluates the predictive strength of all loci by averaging the importance of each in all possible SNP-SNP and SNP-covariate contexts.

In many genetic association studies, a common approach is to follow up the most significant results from a GWAS by genotyping independent samples for a smaller number of SNPs (e.g., 1536 or 3072) using customized arrays [7]. At this point, it is unknown whether RF would be more likely than standard regression methods to include true causative SNPs in the second stage of genotyping in the presence of gene-gene or gene-environment interactions in GWAS data.

To address this issue, the Genetic Analysis Workshop 16 (GAW16) Problem 3 simulated data set was used to evaluate performance of RF and several other association methods. Given knowledge of the major causative SNPs and risk factors for these simulated traits [8], we compared whether the major risk factors were detected in the RF analyses performed in the statistical package R to standard association tests in the computer program PLINK [9], and backward-elimination RF [3], which iteratively eliminates a pre-specified portion of predictors based on low importance values until the error rates of test datasets (out-of-bag samples) minimize to a certain point. Methods were evaluated based on whether the associated SNPs were among the most significant set of SNPs to be selected for genotyping at a hypothetical "second stage".

## Methods

The Framingham Heart Study SHARe dataset for GAW16 Problem 2 formed the basis of the simulated data pedigree structures: 6,476 individuals with both phenotype and genotype data in 942 pedigrees across three generations plus 188 unrelated individuals. The genotypes for all Problem three replicates were fixed to those that were actually observed for the Framingham Heart Study SHARe participants. Phenotypes were simulated for all genotyped individuals at three time points, 10 years apart. Total cholesterol (CHOL) was defined as the sum of the simulated phenotypes triglyceride, high-density lipoprotein (HDL), and low-density lipoprotein (LDL). The traits were simulated such that over time, one develops cranial adipose cumulation (CAC). People with high levels of CAC were at higher risk for myocardial infarction (MI). Smoking also increased the risk of MI in these simulated data. The MI trait was simulated so that the age-adjusted incidence of MI was higher for men than for women. The MI and CAC traits, along with the strongest causative risk factors on the etiologic pathway used in the simulation, are detailed in Kraja et al. [8]. Two hundred replicate datasets were created based on the same generating model.

### Genotype quality control

In the 50 k-SNP panel (48,071 SNPs), quality control measures were performed in the following order: 1) individuals with genotype calls for <95% of the SNPs were removed; 2) all genotypes with a confidence score (the probability that a given genotype is accurate, provided by the BRLLM allele-calling algorithm [10,11]) <0.95 were considered as missing; 3) SNPs with call rates <98% or minor allele frequencies (MAF) <1% were removed. 4) An additional 1,317 SNPs departing from Hardy-Weinberg proportions (*p *< 0.01) were excluded, leaving 31,538 SNPs for the analyses. Markers showing mendelian inconsistencies were coded as unknown in the parent(s).

### Phenotype definition

Phenotype observations from the first two replicates in the simulated data were combined for case and control selection (three phenotype time-points per replicate) using both the family data and the unrelated individuals (singletons) as described below. Because the number of unrelated cases with an MI in any one replicate was small, these two replicates were combined to increase the power to detect variables that contribute to risk of the simulated disease by increasing the number of cases. Subjects were classified as a "case" if they had at least one MI and as a "control" if they were free from any MI over all six time-points. Families were classified as "case-families" if they contained at least one case and "control-families" if they had no cases. Among the genotyped persons, 563 unrelated cases (329 males) and 553 unrelated controls (243 males) were selected. These included all singletons in addition to the youngest case from each case family and the oldest control from each control family. Environmental risk factors (age, sex, smoking, and CHOL) and CAC were taken from the visit that corresponded to the earliest MI for cases and the final visit in the first replicate for controls. Where a case had an MI in both replicates at the same time point, phenotypes from the first replicate were selected. These 1,116 persons were used in all analyses described here.

### RF analyses

Imputation of missing genotypes (1% of genotypes) was performed using the RfImpute function implemented in randomForest package 4.5 in R 2.7.1. RF analyses were run 100 times, randomly sampling 31,000 SNPs in each run with 1000 trees per forest. Each RF model included covariates of age in years, sex, CHOL, and smoking status (yes, no) as potential predictors. Analyses were performed for MI as a dichotomous trait for RF classification and CAC as a quantitative trait for RF regression. Each of these analyses was performed in two ways, using either 200 or 7 randomly sampled predictors at each node (mtry option in the randomForest package) in each tree.

The results over the 100 RFs were summarized by counting the number of times the major causative loci for the CAC and MI traits appeared in the subsets of SNPs identified by each RF as being most predictive based on importance indices generated by randomForest [1]: GINI (the sum over all trees of the decrease in Gini impurity after each split) and mean decrease in accuracy (MDA) for classification or mean decrease in mean squared error (MSE) for regression. These subsets included either 1536 or 3072 SNPs to match the numbers of SNPs often used for second stage genotyping in GWAS studies. Backward-elimination variable selection was performed using the varSelRF 0.6-5 package with the corresponding RF options as described above (1000 trees per forest) but discarding 40% of the lowest ranked predictors at the start of each new forest.

### PLINK analyses

Case-control (Cochrane-Armitage trend) and quantitative-trait (linear regression) association analyses were performed in PLINK [9]. Multivariate linear and logistic regression, adjusting for linear effects of age, sex, smoking status, and CHOL, were also used under additive genetic and log-additive models. For regression analyses, *p*-values were obtained using Wald *t*-tests; for the Cochrane-Armitage test, they were derived from the asymptotic chi-squared distribution (1 df).

## Results and discussion

For comparison with our RF analyses, standard significance tests for association were performed for MI and CAC, adjusting for covariates using PLINK. Among the SNPs that were simulated to have the strongest genetic effects on MI and CAC, none showed genome-wide significance, and only rs12565497 (*p *= 0.003) showed a nominal significance level (MI below 0.01) (Tables 1 and 2). In multivariate linear regression, both sex and CHOL were significantly correlated with CAC. In multivariate logistic regression, age, sex, smoking status, and CHOL were highly associated with MI. The lowest *p*-value among causal markers was *p *= 0.000192 for rs12565497. This SNP ranked 16^th ^among all 31,538 markers tested with multivariate logistic regression. Two other causal SNPs (rs17714718 and rs1894638) would have been retained for second-stage analysis using the univariate rankings (at 1549 and 2683, respectively), but not based on the multivariate analysis.

**Table 1:** PLINK and RF results for MI

	PLINK^a^		RF			
		
	*p*-Value^b^ (rank)		REP^c^ Top 1536		REP^c^ Top 3072	
	
Predictors	Univariate analysis	Multivariate analysis	mtry = 200^d^	mtry = 7^d^	mtry = 200^d^	mtry = 7^d^
Covariates						
Age	-	7.66 × 10^-7^	100	100	100	100
Sex	-	6.97 × 10^-12^	100	36	100	44
Smoking	-	1.72 × 10^-10^	100	71	100	84
Cholesterol	-	<1.0 × 10^-50^	100	100	100	100
	
CAC						
rs6743961 (MAF = 0.49)	0.451 (16568)	0.7394 (24158)	2	10	9	22
						
rs17714718 (MAF = 0.49)	0.0188 (1549)	0.093 (3893)	20	13	33	29
						
rs1894638 (MAF = 0.49)	0.0406 (2683)	0.3198 (11586)	5	18	14	32
						
rs1919811 (MAF = 0.49)	0.2106 (9215)	0.2088 (8004)	8	14	9	21
						
rs213952 (MAF = 0.20)	0.3923 (14807)	0.2319 (8758)	11	3	25	11
	
MI						
rs12565497 (MAF = 0.30)	0.00303 (370)	0.000192 (16)	56	21	71	37
						
rs11927551 (MAF = 0.29)	0.963 (30577)	0.6993 (22956)	1	4	7	9

^a^PLINK analyses assumed either additive or log-additive genetic models.

^b^*p*-Values for covariates were averages across all SNPs.

^c^REP: the number of times in 100 RFs (1000 trees each) in which the given SNP of interest appeared in the top 1536 or 3072 predictors based on GINI index.

^d^mtry: the number of predictors (200 or 7) randomly selected at each node to find the best split while growing trees.

**Table 2:** PLINK and RF results for CAC

	PLINK^a^		RF			
		
	*p*-Value^b^ (rank)		REP^c^ Top 1536		REP^c^ Top 3072	
	
Predictors	Univariate analysis	Multivariate analysis	mtry = 200^d^	mtry = 7^d^	mtry = 200^d^	mtry = 7^d^
Covariates						
Age	-	0.508	100	65	100	65
Sex	-	1.79 × 10^-13^	13	6	19	6
Smoking	-	0.259	11	7	17	7
Cholesterol	-	<1.0 × 10^-50^	100	100	100	100
	
CAC						
rs6743961 (MAF = 0.49)	0.113 (5427)	0.588 (19314)	6	7	11	11
						
rs17714718 (MAF = 0.49)	0.0299 (1940)	0.1392 (5287)	3	5	7	8
						
rs1894638 (MAF = 0.49)	0.0129 (1024)	0.5723 (18983)	4	9	5	12
						
rs1919811 (MAF = 0.49)	0.128 (5948)	0.1413 (5351)	5	5	7	6
						
rs213952 (MAF = 0.20)	0.0786 (4094)	0.4738 (15929)	4	6	8	14

^a^PLINK analyses assumed either additive or log-additive genetic models.

^b^*p*-Values for covariates were averages across all SNPs.

^c^REP: the number of times in 100 RFs (1000 trees each) in which the given SNP of interest appeared in the top 1536 or 3072 predictors based on MSE index.

^d^mtry: the number of predictors (200 or 7) randomly selected at each node to find the best split while growing trees.

In the main RF analyses, we compared several analysis strategies. First, we compared which importance score was best to use for ranking SNPs (MDA vs. GINI (MSE)). We observed that the causal SNPs and important covariates were more likely to be included in the top predictor lists to pass to second-stage analysis when the ranking was based on the GINI index for classification (REP in Table 1) or MSE index for regression (REP in Table 2) than when based on MDA scores. For example, rs12565497 appeared in the TOP 3072 list in 71% of the 100 forests when using GINI compared with 13% using the MDA index (data not shown). It should be noted that Strobl et al. [12] have shown that there is bias in several of the variable-selection processes (including GINI and a permutation accuracy importance measure) often used in RF analyses when there are quantitative traits on different scales or categorical predictors with different numbers of categories, such that non-causative continuous variables and non-causative variables with large numbers of categories are more likely to be selected as important predictor variables than are variables with small numbers of categories. Because no variables with large numbers of categories were used in this study and the only two continuous variables included in the analysis were known to have an effect on the simulated trait, this sort of bias would not be observed here. However, this potential bias should be accounted for in a study of real data, as suggested by Strobl et al. [12]. Results presented here used categorical (genotypic) coding of genotypes, but additive coding gave similar results.

Table 1 shows that for MI, all environmental risk factors were included in the lists of top predictors in 100% of the 100 forests when using the mtry option = 200. For CAC, only age and CHOL ranked highly in all forests. This is reasonable because the simulation framework of MI incorporated smoking status, CAC (and therefore CHOL), and age as risk factors, whereas CAC itself had only age and CHOL as direct risk factors. Age and sex were significantly different in cases and controls (*p *= 2.2 × 10^-16 ^and 1.72 × 10^-6^, respectively). RF performed very well in detecting these environmental risk factors that had comparatively large effects on the traits. RF analyses that included these risk factors had lower mean prediction error rates in 100 RFs (mean = 23% and 40% for mtry = 200 and 7, respectively) than did RF analyses that did not include these covariates (mean = 35% and 43% for mtry = 200 and 7, respectively).

As expected, the number of times that causal SNPs were ranked high enough to be selected for second-stage analysis (top lists) across the 100 RFs increased with the number of SNPs included in the top lists. Only rs12565497, which was simulated to interact with smoking to affect risk of MI [8], had a moderately high frequency (71%) of being in the top 3072 predictors across the 100 RFs. No other SNPs were included in the top 3072 predictors in more than 33% of the 100 RFs (Table 1). None of the five causal SNPs were included frequently in the top 3072 predictors for CAC, indicating very low statistical power to detect association. Using a larger number (mtry = 200) of predictors at each node rather than a small number (mtry = 7) resulted in better performance as measured by the number of times that causal SNPs were included in the top 3072 SNPs across the 100 RFs. Examination of the top 100 predictors over the 100 RFs for each trait revealed that none of these SNPs were closer than 4 Mbp to any of the causative loci and none were in linkage disequilibrium with any of the causative loci, thus showing that proxy SNPs for the causal loci were also not serving as strong predictors in the RF analyses. RF using the backward-elimination algorithm took 1 day per 1 RF, and the lists of top predictors for MI included all environmental risk factors, but none of the causal SNPs (data not shown).

## Conclusions

These RF results supported the use of the GINI (MSE) index rather than MDA, a large number of sampled predictors per node (i.e., mtry option number in randomForest), and categorical coding of genotype data when using RF for the first stage of a GWAS. In this fairly small sample, the strategy of picking random samples of the available SNPs for each RF analysis and then averaging results over 100 forests was not very successful in including the major causative SNPs in the top-ranked sets of SNPs so that they would be included in later replication analyses - only one causal SNP occurred in the top list of SNPs frequently. However, the performance of the RF and regression analyses were very similar and the results suggest that RF may perform well at detecting interactions when the sample size is larger and overall power to detect genetic effects is thus larger. Future simulation studies in much larger samples will be required to resolve this question.

## List of abbreviations used

CAC: Cranial adipose cumulation; CHOL: Cholesterol; GAW: Genetic Analysis Workshop; GINI: Gini index; GWAS: Genome-wide association study; HDL: High-density lipoprotein; LDL: Low-density lipoprotein; MAF: Minor allele frequency; MDA: Mean decrease in accuracy; MI: Myocardial infarction; MSE: Mean squared error; RF: Random forests; SNP: Single-nucleotide polymorphism.

## Competing interests

The authors declare that they have no competing interests.

## Authors' contributions

YK, RW, RAM, HS helped to design the study, perform analyses and draft the manuscript, LW helped design the study and performed analyses, AK, JM, RKL and JEB-W helped design the study and participated in writing the manuscript.

## References

[B1] Breiman L Random forests Machine Learning 2001 45 5 32 10.1023/A:1010933404324

[B2] Bureau A Dupuis J Falls K Lunetta K Hayward B Keith PT Eerdewegh VP Identifying SNPs predictive of phenotype using random forests Genet Epidemiol 2005 28 171 182 10.1002/gepi.20041 15593090

[B3] Diaz-Uriarte R Alvarez de Andres S Gene selection and classification of microarray data using random forest BMC Bioinformatics 2006 7 3 16 16398926 10.1186/1471-2105-7-3 PMC1363357

[B4] Heidema AG Boer MAJ Nagelkerke N Mariman CME Van der ALD Feskens JME The challenge for genetic epidemiologists: how to analyze large numbers of SNPs in relation to complex diseases BMC Genet 2006 7 23 38 16630340 10.1186/1471-2156-7-23 PMC1479365

[B5] McKinney AB Reif MD Ritchie DM Moore HJ Machine learning for detecting gene-gene interactions Appl Bioinformatics 2006 5 77 88 10.2165/00822942-200605020-00002 16722772 PMC3244050

[B6] Lunetta K Hayward L Segal J van Eerdewegh P Screening large scale association study data: exploiting interactions using random forests BMC Genet 2004 5 32 45 15588316 10.1186/1471-2156-5-32 PMC545646

[B7] Perkel J SNP genotyping: six technologies that keyed a revolution Nat Methods 2008 5 447 454 10.1038/nmeth0508-447

[B8] Kraja AT Culverhouse R Daw EW Wu J Van Brunt A Province MA Borecki IB The Genetic Analysis Workshop 16 Problem 3: simulation of heritable longitudinal cardiovascular phenotypes based on actual genome-wide single-nucleotide polymorphisms in the Framingham Heart Study BMC Proc 2009 3 suppl 7 S4 10.1186/1753-6561-3-s7-s4 PMC2795938 20018031

[B9] Purcell S Neale B Todd-Brown K Thomas L Ferreira ARM Bender D Maller J Sklar P de Bakker IW Daly JM Sham CP PLINK: A tool set for whole-genome association and population-based linkage analyses Am J Hum Genet 2007 81 559 575 17701901 10.1086/519795 PMC1950838

[B10] Affymetrix Power Tools (APT) Release apt-1.8.6 http://www.affymetrix.com/support/developer/powertools/changelog/index.html

[B11] Rabbee N Speed TP A genotype calling algorithm for affymetrix SNP arrays Bioinformatics 2006 22 7 12 10.1093/bioinformatics/bti741 16267090

[B12] Strobl C Boulesteix AL Zeileis A Hothorn T Bias in random forest variable importance measures: illustrations, sources and a solution BMC Bioinformatics 2007 8 25 46 17254353 10.1186/1471-2105-8-25 PMC1796903

